# Effect of Elevated Carbon Dioxide Exposure on Nutrition-Health Properties of Micro-Tom Tomatoes

**DOI:** 10.3390/molecules27113592

**Published:** 2022-06-02

**Authors:** Linda Boufeldja, Dennis Brandt, Caroline Guzman, Manon Vitou, Frederic Boudard, Sylvie Morel, Adrien Servent, Claudie Dhuique-Mayer, Léa Ollier, Orianne Duchamp, Karine Portet, Christian Dubos, Patrick Poucheret

**Affiliations:** 1 Qualisud, CIRAD, IRD, Institut Agro, Université Montpellier, Avignon Université, Université de La Réunion, 34398 Montpellier, France; boufeldjalinda@gmail.com (L.B.); caroline.guzman@umontpellier.fr (C.G.); frederic.boudard@umontpellier.fr (F.B.); adrien.servent@cirad.fr (A.S.); claudie.dhuique-mayer@cirad.fr (C.D.-M.); lea.ollier@cirad.fr (L.O.); orianne.duchamp@gmail.com (O.D.); karine.portet@umontpellier.fr (K.P.); 2 IPSiM, CNRS, INRAE, Institut Agro, Université de Montpellier, 34000 Montpellier, France; dennis.brandt@uni-muenster.de (D.B.); christian.dubos@inrae.fr (C.D.); 3 Laboratoire de Botanique, Phytochimie et Mycologie, CEFE, CNRS, Université de Montpellier, Université Paul-Valéry Montpellier-EPHE-IRD, 34000 Montpellier, France; manon.vitou@umontpellier.fr (M.V.); sylvie.morel@umontpellier.fr (S.M.)

**Keywords:** micro-tom tomato, elevated atmospheric carbon dioxide, nutrition, health

## Abstract

(1) Background: The anthropogenically induced rise in atmospheric carbon dioxide (CO_2_) and associated climate change are considered a potential threat to human nutrition. Indeed, an elevated CO_2_ concentration was associated with significant alterations in macronutrient and micronutrient content in various dietary crops. (2) Method: In order to explore the impact of elevated CO_2_ on the nutritional-health properties of tomato, we used the dwarf tomato variety Micro-Tom plant model. Micro-Toms were grown in culture chambers under 400 ppm (ambient) or 900 ppm (elevated) carbon dioxide. Macronutrients, carotenoids, and mineral contents were analyzed. Biological anti-oxidant and anti-inflammatory bioactivities were assessed in vitro on activated macrophages. (3) Results: Micro-Tom exposure to 900 ppm carbon dioxide was associated with an increased carbohydrate content whereas protein, minerals, and total carotenoids content were decreased. These modifications of composition were associated with an altered bioactivity profile. Indeed, antioxidant anti-inflammatory potential were altered by 900 ppm CO_2_ exposure. (4) Conclusions: Taken together, our results suggest that (i) the Micro-Tom is a laboratory model of interest to study elevated CO_2_ effects on crops and (ii) exposure to 900 ppm CO_2_ led to the decrease of nutritional potential and an increase of health beneficial properties of tomatoes for human health.

## 1. Introduction

The rise in atmospheric carbon dioxide (CO_2_) and associated climate change are clearly attributed to human activities. Before the industrial development era, CO_2_ concentration was 280 ppm (parts per million). In 2021, the average atmospheric concentration in carbon dioxide recorded has reached 410 ppm and is predicted to increase up to 750–1300 ppm by the year 2100 [1]. This was expected, as the physiological model suggested, to stimulate carbon fixation thereby increasing biomass production and yield [2]. However, FACE (Free-Air CO_2_ Enrichment) experiments on crops (C3 species such as wheat, rice, …) provided data suggesting a significant deviation when compared to plant physiological model predictions. Elevated CO_2_ (eCO_2_) was associated with a lower increase in biomass production, and plants’ mineral status appeared compromised with reduced concentration of iron and nitrogen [3]. This phenomenon, known as CO_2_ acclimation in response to eCO_2_, is characterized by carbohydrate accumulation in plants and repression of photosynthetic efficiency. It exacerbates mineral nutrient limitation on vegetal growth and reduction of nutrients concentration in plants [4]. These observations were generalized to many other plant nutrients such as iron and most vitamins [5,6,7]. Retrospective data analysis tended to confirm this phenomenon since contemporary plants (410 ppm CO_2_) demonstrated lower nutrient status than their pre-industrial era counterparts (280 ppm CO_2_) [5]. The underlying mechanisms leading eCO_2_ to nutrient limitation of vegetal growth and decreased nutrient concentration on plants are not established. This negative effect on both plant nutrition and plant nutritional profile may not be only explained by a dilution effect associated with the larger biomass [8]. Therefore, eCO_2_ would have an unexpected *per se* negative impact on plant nutrition physiology and nutritional profile [9,10].

The pre-cited effects of eCO_2_ also affect crops such as tomatoes. Molecular content, as well as antioxidant properties, were recorded to be altered. As a function of the condition of growth and eCO_2_ concentration, eCO_2_ was associated with the accumulation of carbohydrates and decreased protein content in relation to nitrogen plant metabolism alteration. Fruit content in minerals such as iron (Fe), magnesium (Mg), and zinc (Zn) was reduced. Indeed, one could propose that part of the negative impact of eCO_2_ on nitrogen (protein) and mineral content, such as iron, in crops such as tomato could be overcome by increasing nutrient inputs in the soil through the use of fertilizers. Obviously, such an approach is not suitable to maintain the nutritional value of crops, as sustainable agriculture aims at developing environmentally-friendly practices. Therefore, it is also important to investigate how plant nutrient limitation interacts with eCO_2_ to modulate crops’ nutrition-health properties. The influence of eCO_2_ on carotenoids and more specifically lycopene, was variable with a potential influence of temperature on the synthesis of this compound. The underlying physiological, genetic, and molecular mechanisms of these modifications remain to be elucidated [11].

Therefore, it was demonstrated, on various plants including tomatoes, that eCO_2_ alters plants’ ionome as well as macronutrients (carbohydrates and proteins), micronutrients, and vitamins contents. [5,6,12]. A higher carbohydrate/protein ratio and an increased level of anti-nutrients in staple food crops were also confirmed. For these reasons, eCO_2_ was considered a potential threat to human nutrition [13] and it may favour not only nutritional disorders but also chronic metabolic diseases thereby potentially becoming a major public health issue worldwide in the upcoming future. Modification of plant composition due to differential pedoclimatic conditions is known to significantly influence bioactive molecular ratio, associated bioactivity, and therefore nutrition and health properties [14,15]. In the case of eCO_2_, the major alterations of plants, and more specifically of tomato, in macro- and micronutrient content may have potentially major impacts on their nutrition-health value. Higher carbohydrate content combined with lower protein, minerals, and vitamins represent a potential impairment of nutrition-health value for humans that may contribute: (1) to nutritional deficiencies in protein and micronutrients as well as (2) to nutritional excess in carbohydrates increasing the risk factors of chronic non-communicable diseases (NCDs) such as metabolic syndrome, diabetes and cardiovascular disease (CVD). The latter is associated with low-grade inflammation and excessive platelet activation contributing to atherosclerosis and prothrombotic status thereby increasing the risk of CVD complications and co-morbidities [16,17]. Such drift in crops, like tomato, could compromise food transition toward sustainable agri-food production systems and ultimately consumer health.

Tomato (*Solanum lycopersicum* L.) is a major crop in economical and food terms. It is a source of nutrients and bioactive molecules including carotenoids, phenolic compounds, vitamins, and minerals [18]. These compounds are associated with several individual and synergistic beneficial health effects. Indeed, tomato phytochemistry integrates bioactive compounds whose combinations are known to influence various inflammatory molecular signaling pathways involved in CVD through platelet activity modulation [19,20,21]. Any modification in the composition profile of tomato might hinder its nutritional and health value [22]. Among the numerous variety of tomato, the dwarf tomato *Solanum lycopersicum* cv. Micro-Tom is used as a plant model. Nonetheless, Micro-Tom response to eCO_2_ in terms of fruit composition and nutrition-health properties was never investigated.

The objective of the present study was to produce a Micro-Tom tomato model under 400 ppm versus 900 ppm atmospheric CO_2_ conditions in growing chambers under nutritional constraints, nitrogen and/or iron deficiency. These two plant mineral nutrients were chosen since it has been reported that their accumulation in tomato is inhibited by eCO_2_. In addition, they play a key role in crop productivity, quality of their derived products, and are frequently a limiting resource in soils. Minerals and phytochemical composition as well as biological properties, i.e., antioxidant and anti-inflammatory immunomodulatory bioactivities, were assessed to evaluate the impact of predicted CO_2_ elevation on the nutrition-health potential of tomato.

## 2. Results

### 2.1. Chemical Composition

#### 2.1.1. Macronutrients Content

**Figure 1:**
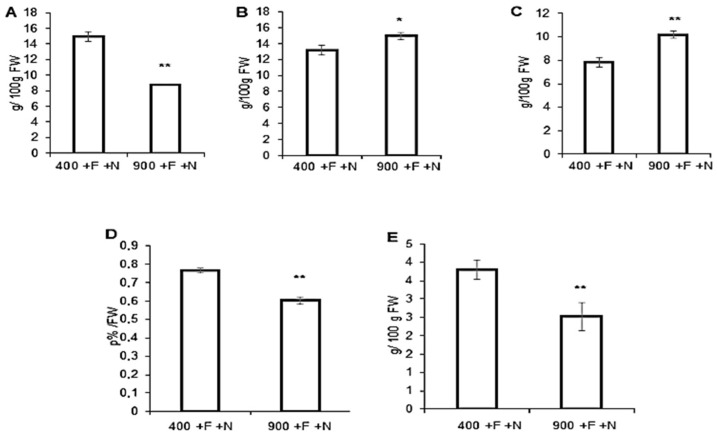
**Macronutrient content of Micro-Tom tomato fruits.** Fruits samples were collected from hydroponically grown plants (Control condition: +F/+N) under ambient (400) ppm or elevated (900 ppm) atmospheric CO_2_ concentrations. (**A**) Sucrose, (**B**) Fructose, (**C**) Glucose, (**D**) Total proteins, (**E**) Total lipids. *T*-test significance: *, *p* < 0.05; **, *p* < 0.01.

Results of Micro-Tom tomatoes macronutrients content are presented in Figure 1. Fruits samples were collected from hydroponically grown plants. The control condition with iron, Fe, and nitrogen, N, (i.e., +F/+N) is reported in Figure 1 under ambient (400 ppm) or elevated (900 ppm) atmospheric carbon dioxide (CO_2_). Data indicated that Micro-Tom cultivated under elevated CO_2_ concentrations (eCO_2_) contained a significantly lower concentration of sucrose, total proteins, and total lipids. Conversely, the same fruits contained a significantly higher concentration of fructose and glucose. From a statistical point of view, the major differences were recorded for sucrose, total protein and lipids (reduction), and glucose (elevation).

#### 2.1.2. Micronutrients

##### Minerals Content

**Figure 2:**
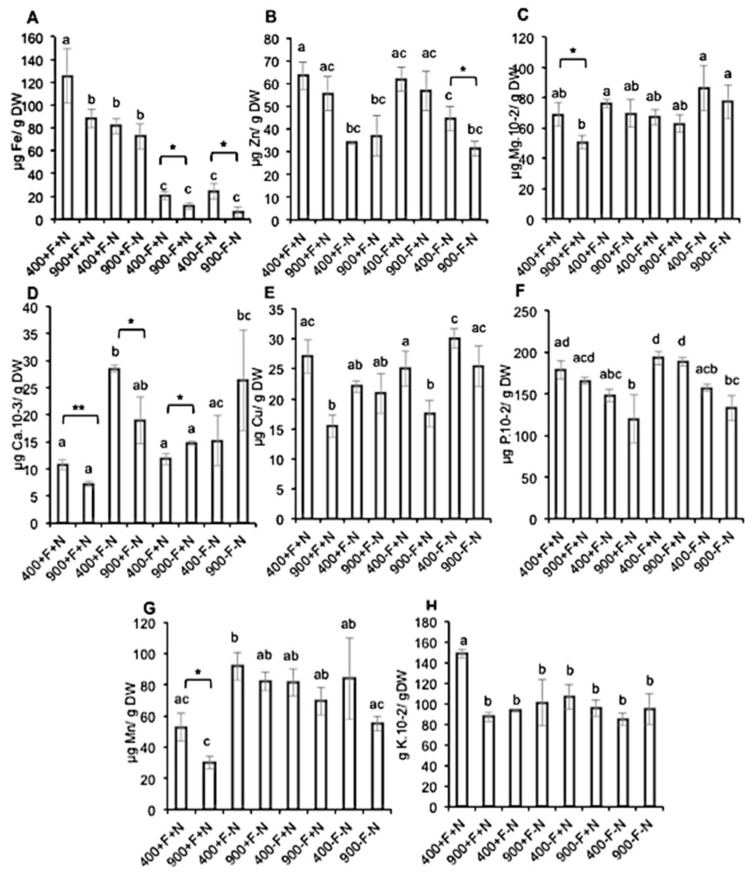
**Mineral nutrient content of Micro-Tom tomato fruits.** Fruit samples were collected from hydroponically grown plants under ambient (400 ppm) or elevated (900 ppm) atmospheric CO_2_ concentrations with four nutritional regimes: control (+F/+N), iron deficiency (−F/+N), nitrogen deficiency (+F/−N) or dual deficiency (−F/−N). (**A**) Iron, (**B**) Zinc, (**C**) Magnesium, (**D**) Calcium, (**E**) Copper, (**F**) Phosphorus, (**G**) Manganese, (**H**) Potassium. Error bars show ±SD (*n* = 3). *T*-test significance: *, *p* < 0.05; **, *p* < 0.01. Means within each condition with the same letter are not statistically different according to one-way ANOVA followed by post hoc Tukey test (*p* < 0.05).

The concentration of eight mineral nutrients was measured, namely iron (Fe), zinc (Zn), magnesium (Mg), calcium (Ca), copper (Cu), phosphorus (P), manganese (Mn), and potassium (K) (Figure 2). Overall, the accumulation of these eight mineral nutrients in fruits of plants grown in control (+F/+N) condition was decreased in response to eCO_2_ exposure. These observations were in agreement with previous studies [13].

In contrast, when the plants were grown under nitrogen limiting conditions (+F/−N), the impact of eCO_2_ exposure was limited. Actually, a significant decrease in mineral nutrient concentrations in response to eCO_2_ exposure was only observed for Ca. However, this trend, even if not significant, was also observed for Fe, Mg, P, and Mn. Zn and K concentrations were reduced in response to nitrogen deficiency whereas those of Ca and Mn were increased when compared to the Control (+F/+N) condition.

When grown under iron limiting conditions (−F/+N), once again, the impact of eCO_2_ exposure was limited. A significant decrease upon eCO_2_ exposure was only observed for Fe and Cu concentrations. A similar trend was measured for Mn and K. As expected, a strong reduction in Fe concentration in response to iron deficiency was observed when compared to the control (+F/+N) condition. K content was affected in a similar manner in response to iron deficiency. In contrast, Ca content was increased in response to eCO_2_ exposure.

Under the dual deficiency (−F/−N) condition, eCO_2_ exposure led to a significant decrease accumulation of Fe, Zn, Cu, and P. Fe, Zn, and K content was decreased in response to the dual nitrogen and iron deficiency when compared to the control (+F/+N) condition.

##### Total Carotenoids and Lycopene Contents

**Figure 3:**
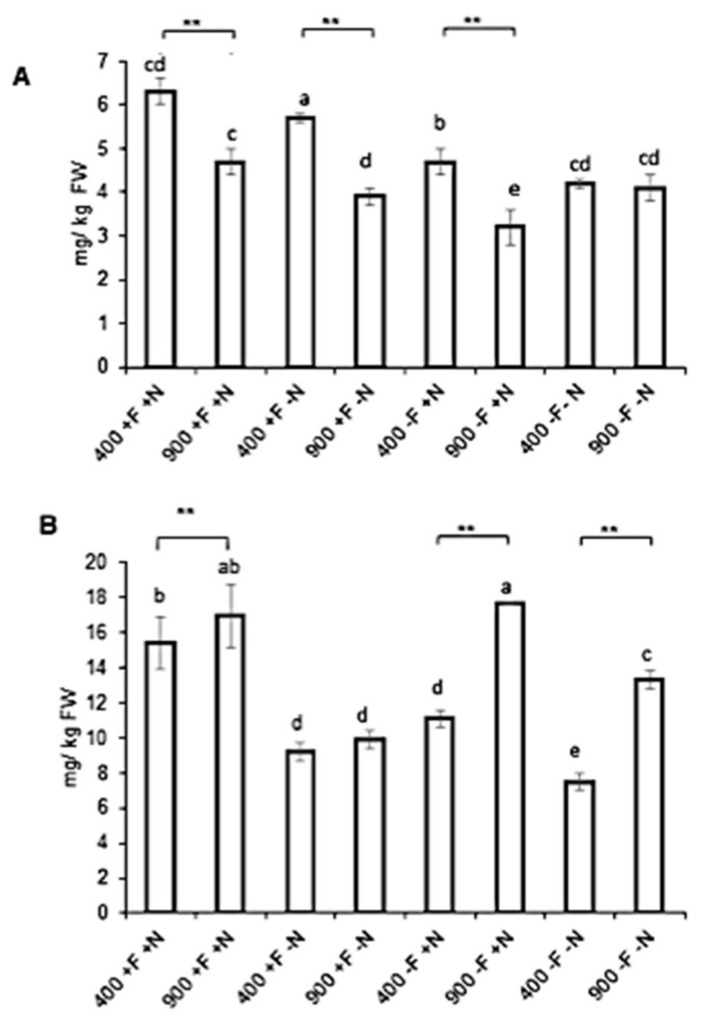
**Carotenoids content of Micro-Tom tomato fruits.** Fruits samples were collected from hydroponically grown plants under ambient (400 ppm) or elevated (900 ppm) atmospheric CO_2_ concentrations with four nutritional regimes: control (+F/+N), iron deficiency (−F/+N), nitrogen deficiency (+F/−N) or dual deficiency (−F/−N). (**A**) Total carotenoids content, (**B**) Lycopene content. Error bars show ±SD (*n* = 3). *T*-test significance: **, *p* < 0.01. Means within each condition with the same letter are not statistically different according to one-way ANOVA followed by post hoc Tukey test (*p* < 0.05).

Dosages of Micro-Tom tomatoes carotenoids content are presented in Figure 3. Fruits samples were collected from hydroponically grown plants. On the control condition (+F/+N), eCO_2_ exposure was associated with a decreased content in total carotenoids. The same impact was also recorded for the two regimens, (+F/−N) and (−F/+N) but not for the most deprived (−F/−N). For the latter, no difference was observed between ambient CO_2_ and eCO_2_. Iron and nitrogen seemed to influence carotenoid content independently from each other since when either nutrient alone was not present, i.e., (+F/−N) or (−F/+N), carotenoids level was decreased, when compared to control. In the case of both iron and nitrogen concentration being decreased (−F/−N) in the growth media, carotenoids level was unaffected. Regarding lycopene content, the control condition (+F/+N), when exposed to eCO_2_, was characterized by an increased level of lycopene. The same impact was recorded for the conditions (−F/+N) and (−F/−N) but not for (+F/−N) where eCO_2_ did not seem to influence lycopene content. In addition, lycopene content under ambient CO_2_ seemed to be affected by nutrients. Indeed, when iron or nitrogen or both were withdrawn, lycopene concentration was decreased.

### 2.2. Antioxidant Bioactivity

**Figure 4:**
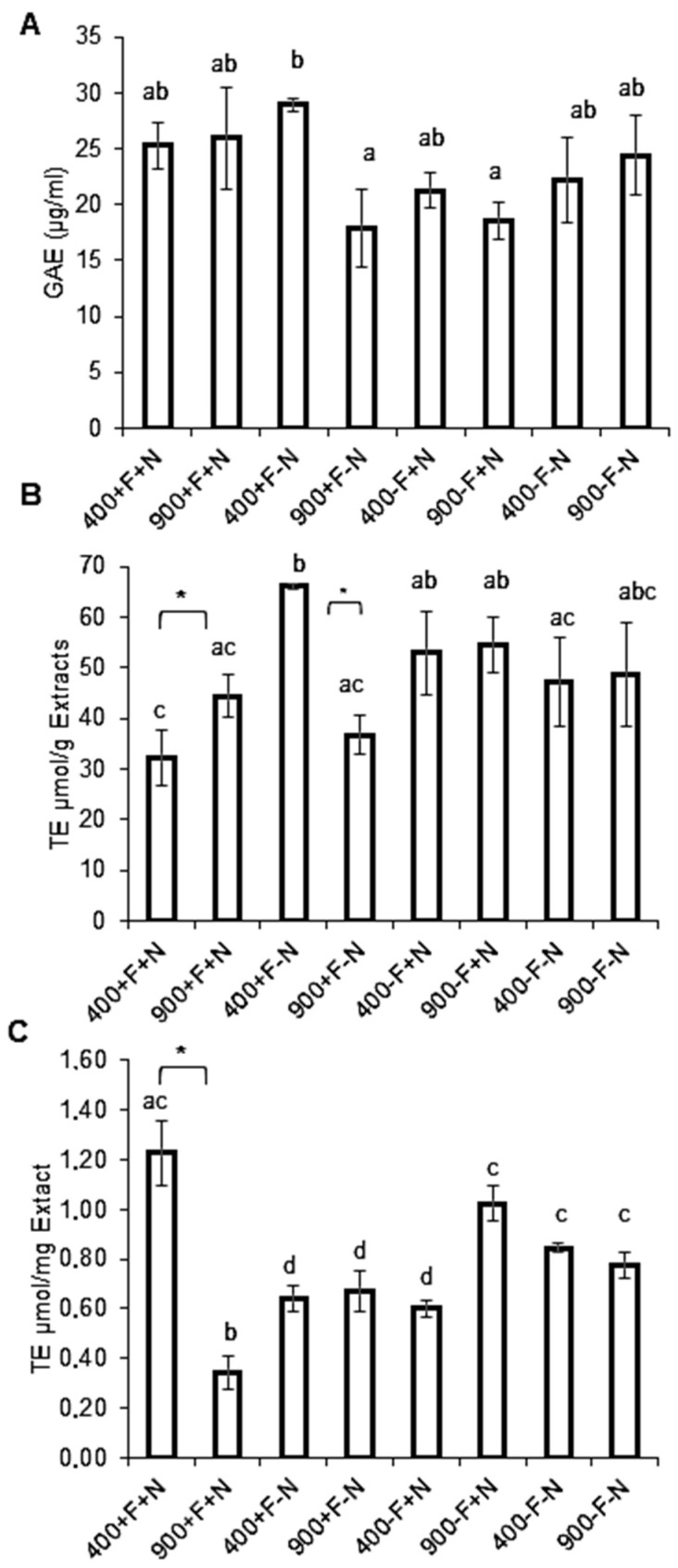
**Antioxidant activity of Micro-Tom tomato fruits extracts.** Fruits samples were collected from hydroponically grown plants under ambient (400 ppm) or elevated (900 ppm) atmospheric CO_2_ concentrations with four nutritional regimes: control (+F/+N), iron deficiency (−F/+N), nitrogen deficiency (+F/−N) or dual deficiency (−F/−N). (**A**) Total polyphenol content (Folin–Ciocalteu method), (**B**) 2,2-diphenyl-1-picrylhydrazyle (DPPH) assay, (**C**) Oxygen radical absorbance capacity (ORAC) assays. Error bars show ±SD (*n* = 3). *T*-test significance: *, *p* < 0.05. Means within each condition with the same letter are not statistically different according to one-way ANOVA followed by post hoc Tukey test (*p* < 0.05).

Total polyphenol content (TPC) of Micro-Tom extracts (Figure 4a) was not affected by eCO_2_ exposure in Control (+F/+N) and in (−F/+N) or (−F/−N) conditions. Only the (+F/−N) regimen was associated with a significantly decreased TPC associated with eCO_2_ exposure. On the other hand, when considering ambient CO_2_ exposure or eCO_2_ independently, nutrients (iron or nitrogen) did not seem to influence TPC within each condition. To assess the potential correlation of TPC with antioxidant capacity, Micro-Tom extracts were submitted to DPPH and ORAC tests.

DPPH results (Figure 4b) indicated that in the (+F/+N) condition, eCO_2_ induced an increased free radical scavenging capacity. Conversely, eCO_2_ exposure was associated with a decreased scavenging potential under the (+F/−N) regime. The others regimen, in both CO_2_ conditions, were unaffected.

ORAC results (Figure 4c) showed that eCO_2_ majorly decreased the antioxidant capacity of Micro-Tom extracts under the control regime (+F/+N). This effect was the opposite when iron was withdrawn (−F/+N). Within ambient CO_2_ conditions, withdrawal of nutrients was associated with a decrease in antioxidant capacity. Oppositely, under eCO_2_ conditions, withdrawal of nutrients was associated with an increased antioxidant capacity.

### 2.3. Anti-Inflammatory—Immunomodulatory Bioactivity

#### 2.3.1. Cell Viability

Cell exposure to Micro-Tom extracts did not alter macrophages viability thereby allowing exploring anti-inflammatory activity without adverse influence (data not shown).

#### 2.3.2. Nitric Oxide Production

**Figure 5:**
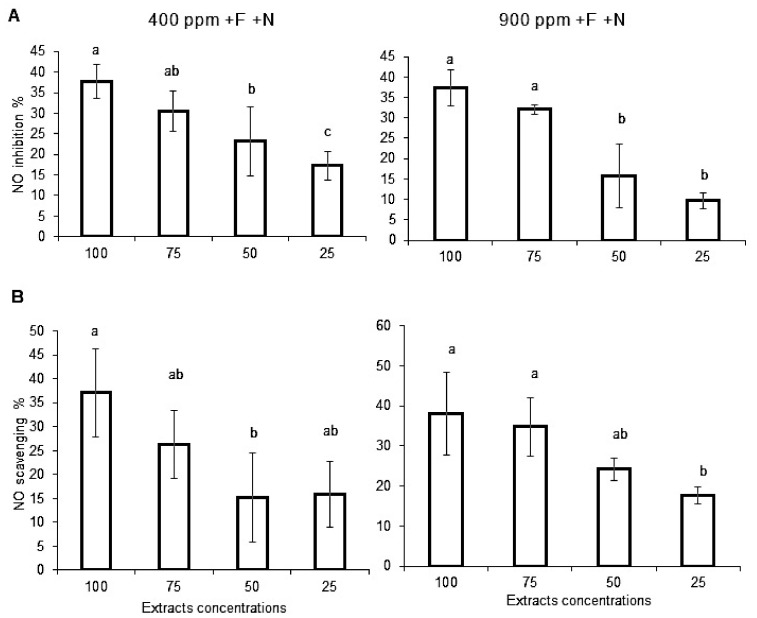
**Effect of Micro-Tom tomato fruits extracts on nitric oxide liberation and scavenging capacity.** Fruits samples were collected from hydroponically grown plants (Control condition +F/+N) under ambient (400 ppm) or elevated (900 ppm) atmospheric CO_2_ concentrations. (**A**) Fruits extract inhibition rates on nitric oxide production by J774 macrophages cells stimulated with lipopolysaccharide (LPS) and interferon gamma (IFNγ), (**B**) Fruits extract nitric oxide scavenging capacity. Error bars show ±SD (*n* = 3). *T*-test significance. Means within each condition with the same letter are not statistically different according to one-way ANOVA followed by post hoc Tukey test (*p* < 0.05).

Nitric oxide (NO) production by stimulated macrophage cells treated with Micro-Tom extracts is presented in Figure 5a. In both ambient CO_2_ and eCO_2_ conditions, Micro-Tom extracts induced a similar (non-statistically different) dose-dependent inhibition of NO production.

To further explore Micro-Tom extracts’ potential bioactivity against NO free radical, the extracts NO-scavenging capacity was assessed. Similarly, in both ambient CO_2_ and eCO_2_ conditions, Micro-Tom extracts demonstrated a dose-dependent NO-scavenging capacity (non-statistically different between the two CO_2_ conditions).

#### 2.3.3. Cytokines Interleukin-6, TNF-α and Prostaglandin-E2

**Figure 6:**
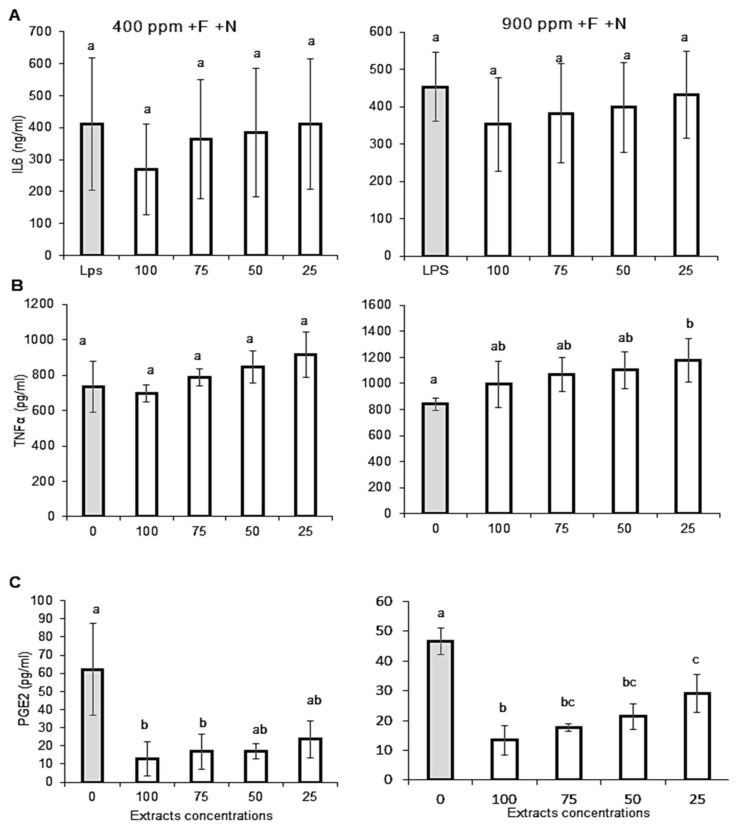
**Effect of Micro-Tom tomato fruits extracts on nitric oxide production and scavenging capacity.** Fruits samples were collected from hydroponically grown plants (Control condition +F/+N) under ambient (400 ppm) or elevated (900 ppm) atmospheric CO_2_ concentrations. (**A**) Interleukin-6 (IL-6), (**B**) Tumour necrosis factor alpha, (**C**) Prostaglandin E2 (PGE-2) levels were measured by ELISA in the supernatant of J774 macrophages stimulated with lipopolysaccharide (LPS) and interferon gamma (IFNγ)Error bars show ±SD (*n* = 3).The grey bars refer to control cells activated by LPS/interferon. Means within each condition with the same letter are not statistically different according to one-way ANOVA followed by post hoc Tukey test (*p* < 0.05).

Interleukin-6 (IL-6), TNF-α, and Protaglandin-E2 (PGE-2) productions by stimulated macrophage cells treated with Micro-Tom extracts are presented in Figure 6. In both ambient CO_2_ and eCO_2_ conditions, Micro-Tom extracts did not statistically affect IL-6 production (Figure 6A); even if the graphs could suggest a possible dose-dependent inhibition tendency. Regarding TNF-α production (Figure 6B), it was unaffected under ambient CO_2_ and slightly increased under eCO_2_. Conversely, Micro-Tom extracts significantly decreased PGE-2 production under both CO_2_ and eCO_2_ conditions with a dose dependency for eCO_2_ conditions (Figure 6C).

## 3. Discussion

The literature suggested that “*more research on the interactive effects of eCO_2_ and growth conditions of tomato fruits quality is needed*” [11]. Indeed, few reports explored the influence of the combination of elevated carbon dioxide and various nutrient regimes on tomato fruit composition and nutrition-health properties. The present study was therefore undertaken on the well-known dwarf Micro-Tom tomato plant model.

Micro-Tom produced in growing chambers under an eCO_2_ (900 ppm) atmosphere were characterized, in the control regime group (+F/+N) by an increased content in glucose and fructose but a lower content in protein and lipids. These results are in accordance with previous reports on other types of tomato [23]. Sucrose is the main photo-assimilable disaccharide generated at the level of plant leaves. Upon production, it is transported in the fruits to be cleaved and stored in the form of glucose and fructose monosaccharides. The sucrose gradient between leaves and fruits would modulate sucrose import in the fruits. One possible interpretation of our results may therefore be a potential direct or indirect influence of eCO_2_ on the gradient and/or the enzymes, such as sucrose-synthase, involved in the sucrose import into fruits [24]. Conversely, protein content was clearly decreased in response to eCO_2_ exposure. This observation was also made by other research groups on various crops including tomato [3,6]. Elevation of atmospheric CO_2_ and associated photosynthesis were reported to increase the carbon to nitrogen ratio. It supports the observation of an increased carbohydrate (glucose and fructose) and a decreased protein contents [25]. Considering these elements and the fact that exposure to elevated CO_2_ was reported to increase photosynthesis and biomass, it, therefore, suggests that Micro-Tom fruits’ nutritional quality seems to be negatively altered [23,26]. Indeed, lower food supply in protein associated with a higher supply of glucose and fructose may contribute to the already high incidence of non-communicable metabolic diseases worldwide [27]. In addition, we observed that total carotenoids were decreased but lycopene increased. Carotenoids represent a group of molecules including compounds such as lycopene, lutein, cryptoxanthin, and neoxanthin. They were reported to have significant beneficial health effects. Indeed carotenoid consumption was correlated to lower the risk of metabolic syndrome and associated co-morbidities, i.e., obesity, diabetes, hypertension, cardiovascular diseases, atherosclerosis, and some forms of cancer. In consequence, tomato carotenoids are considered of significant interest as a dietary segment of global food intake [28]. Therefore, the reduction of Micro-Tom total carotenoids content might be considered a negative signal in terms of nutrition-health. Nonetheless, our results also indicated a higher level of lycopene. This data suggests that eCO_2_ exposure may not be completely detrimental. This molecule was demonstrated to bear antioxidant (highest activity among carotenoids), as well as anti-inflammatory effects of interest in metabolic diseases and is of interest in prostate cancer [27]. Indeed, on metabolic syndrome and comorbidities, lycopene was reported to improve carbohydrate and lipid homeostasis through increased insulin-sensitivity, adiponectine levels and dyslipidemia modulation (increased High-Density Lipoprotein, HDL). In addition, more specific bioactivities of lycopene include the inhibition of tumor cell proliferation through enzyme inhibition as well as tumor cell apoptosis induction and epigenetic gene modulations [29]. Based on its combined health effects, lycopene was recognized as “*the only dietary supplement presenting statistically significant association with lower risk for cancer death and all-cause of death*” [30].

When considering plants’ nutritional regimes’ influence on carotenoids content, all regimes were identically impacted by eCO_2_ exposure except the most deprived (−F/−N). The latter demonstrated an absence of difference between the ambient CO_2_ and eCO_2_ exposures suggesting that the combined presence of iron and nitrogen is necessary to partially compensate for the negative effect of elevated carbon dioxide. At the level of lycopene, it appeared that the presence of nitrogen was globally associated with higher lycopene content in ambient CO_2_ and eCO_2_ groups. Conversely, iron deprivation seemed to favor lower lycopene levels (excepted for (−F/+N)). These results suggest that iron and/or nitrogen modulated lycopene content. This observation is in accordance with previous reports on tomato [30] but in disagreement with others [11]. This discrepancy is of great interest as it suggests at least two levels of complexity, (i) the complex interplay of the various mineral plant nutrients on its metabolism and (ii) the differential impact of each type of combination of nutrient on each single metabolite content.

In addition, total glucose was reported, by Rangaswamy et al. [31] to be enhanced by 700 ppm CO_2_. This intermediary exposure, when compared to our study, was associated with better yield and qualitative fruit quality traits. This improvement was compromised by a 2 °C temperature increase. Our results indicate that a further elevation of CO_2_ up to 900 ppm generates similar results to Rangaswamy et al. when considering sugar content [31]. If we did not assay the impact of temperature, we investigated protein and biological potential. Based on the results of Rangaswamy et al., and ours, it appears that in addition to production yield and standard quality traits it might be necessary to include additional parameters (e.g., protein and lycopene content) in determining the actual quality of tomato. It may also be of interest in exploring the effects of the various combinations of temperature, CO_2_ exposure, and plant nutrients on the future nutritional properties of tomato submitted to climate change.

Total polyphenol content (TPC) is a parameter of interest as a first step to assess the putative antioxidant potentiality of a vegetal matrix. In the case of Micro-Tom, our results indicate no significant influence of eCO_2_ exposure (except for the (+F/−N)) or nutrient regime. Micro-Tom polyphenol content was in the average tomato species [32]. Polyphenols are a large family of bioactive plant compounds including subgroups such as phenolic acids and flavonoids (e.g., flavonols, anthocyanins, tannins). Their consumption was recognized for their health benefits in various diseases such as, but not limited to, metabolic pathology, their co-morbidities and cancers. The main effects of these compounds include antioxidant, anti-inflammatory, immunomodulatory and improvement of various pathological conditions, i.e., insulin resistance, central obesity, vascular dysfunctions, dyslipidemia or hyperglycemia [32]. The influence of eCO_2_ exposure may have or not have an impact on polyphenols content depending on the plant types and cultivars [33,34]. It, therefore, appeared that Micro-Tom belongs to plants whose polyphenol content may not be influenced by eCO_2_. To our knowledge, this is the first time that evaluation of Micro-Tom and more generally tomato polyphenol content is measured under eCO_2_ exposure. This observation will need to be replicated on various other cultivars in order to confirm that it is a general property of tomatoes. Furthermore, growth conditions combinations (temperature, nutrients, and atmospheric CO_2_, …) might be an important factor inducing qualitative and quantitative variations of the phytochemical content [34,35]. The possibility to orient tomatoes content brought authors to consider tomato as bearing the potential to be a nutraceutical crop [36]. This concept is further supported by the joint accumulation of polyphenols and carotenoids whose combination in this fruit could explain its significant bioactivity on health [35].

Micro-Tom antioxidant activity, measured by DPPH assay, was increased by eCO_2_ in control condition (+F/+N). This result was reversed by nitrogen withdrawal. In addition, nitrogen withdrawal increased the antioxidant properties of Micro-Tom only at ambient CO_2_. ORAC results showed a major drop in antioxidant activity in the control condition (+F/+N) exposed to eCO_2_. The other regimes including iron and/or nitrogen withdrawal globally decreased antioxidant potential under both CO_2_ conditions. Taken together, DPPH and ORAC results demonstrate the antioxidant potential of Micro-Tom. They also suggest that the antioxidant profile of Micro-Tom is probably influenced by additional factors in addition to polyphenol content since TPC and antioxidant activities are not fully correlated. Literature reported both synchrony and possible asynchrony between TPC and antioxidant potential [34,35]. Several hypotheses can be proposed without being exhaustive. In addition to regime and CO_2_ exposure, factors influencing the antioxidant profile may include carotenoids, and other secondary metabolites but also the multiple qualitative and quantitative possible combinations and ratios of molecules through a complex interplay of synergies and antagonisms [37].

Micro-Tom anti-inflammatory potential was assessed on stimulated macrophage cells. The extracts at both CO_2_ exposure demonstrated similar concentration-dependent anti-inflammatory effects on inflammation markers NO and PGE-2. The inflammation reaction is the first nonspecific response of the immune system associated with macrophages polarization into a pro-inflammatory status. They release inflammation mediators such as NO (vasodilation) and PGE-2 (immune reaction) among others [37,38]. These disturbances are involved in low-grade inflammation known to promote various metabolic diseases [39]. Our results with Micro-Tom indicate a specific anti-inflammatory profile centered on NO and PGE-2 suggesting a potential modulatory role in combined early stage of inflammation, i.e., chemotaxis and vasomotricity. In addition, lycopene is known to inhibit radical oxygen species production and thereby NFkB (Nuclear Factor kappa-light-chain-enhancer of activated B cells) pathway activation as well as inflammatory markers production. Therefore, MicroTom tomato immunomodulatory underlying mechanism of action might involve the combined effects of lycopene on NFkB, phenolic acids on cycloxygenase and cytokine production as well as flavonoids on macrophage functions [40]. Finally, at the organism level, in relation to inflammatory associated diseases, such as metabolic syndrome (MetS) or cardiovascular diseases (CVD), tomato FruitFlow was recognized as an effective anti-inflammatory functional food for prophylactic and/or adjunctive care in systemic low grade inflammatory pathophysiological states (e.g., CVD, MetS) through modulation of excessive platelet function [41,42]. Our results are in general agreement with this underlying potential of tomato. Of course, the regulation of inflammatory processes and the combinations of tomato compounds are so complex and intricated that in-depth molecular investigations will be necessary to better understand the mechanism of action of tomato phytochemicals on the regulation of macrophages responses. Additionally, a general meta-analysis of all results from the literature obtained so far may help identify the precise correlations between the secondary metabolites variations and the recorded bioactivity changes.

## 4. Materials and Methods

### 4.1. Plant Material

100 seeds of tomato variety *Solanum licopersicum* L. cv Mico-Tom were sterilized according to the method described by Appenroth et al. [43]. Briefly, seeds were treated with 0.7% bleach then washed with sterile water 3–4 times and left in water for 4–7 h. Then germination was carried out in Petri dishes containing solid MS2/2 (Murashige and Skoog: 5% *v*/*v* macro-element solution 10× (Sigma-Aldrich, Oslo, Norway, AS M0654), 50 μM KCl, 30 μM H_3_BO_3_, 5 μM MnSO_4_ (hydrate), 1 μM ZnSO_4_ (heptahydrate), 1 μM CuSO_4_ (pentahydrate), 0.1 μM (NH_4_)_6_Mo_7_O_24_ (tetrahydrate), 10 μM Na-Fe-EDTA, 1% *w*/*v* sucrose, 0.05% *w*/*v* MES, 0.7% *w*/*v* agar, pH 5.7.) medium for 13 days. After germination, the plants were transferred in dark and opaque boxes (4 plants per box) and grown in Hoagland medium (hydroponics). The media used was as follow: 1 mM KH_2_PO_4_, 1 mM MgSO_4_, 0.25 mM K_2_SO_4_, 0.25 mM CaCl_2_, 0.5 (−N) or 5 (+N) mM KNO_3_; 10 μM Na-Fe-EDTA, 50 μM KCl, 30 μM H_3_BO_3_, 5 μM MnSO_4_ (hydrate), 1 μM ZnSO_4_ (heptahydrate), 1 μM CuSO_4_ (pentahydrate), 0.1 μM (NH_4_)_6_Mo_7_O_24_ (tetrahydrate). The concentration of iron was 10 μM Na-Fe-EDTA (+F). The boxes were kept in culture chambers under the following conditions: humidity 65%, temperature 20 °C, long day (16 h of light), and two different concentrations of CO_2_ (400 ppm and 900 ppm). Media was changed twice a week. After one month of growth, half of the plants were grown in the absence of iron (−F) giving rise to 4 growth conditions: control (+N/+F) condition, nitrogen deficiency (−N/+F) condition, iron deficiency (+N/−F) condition, and dual deficiency (−N/−F) condition. At this stage, only two plants were retained per box. After 40 days of cultivation in the growth chambers, KNO_3_ concentration was increased from 5 mM to 7.5 mM. Ten days later (day 50) the first fruits appeared. The first harvest of fruits (red stage) was carried out at the beginning of the third month (90 days) and continued until the first days of the fourth month (120 days).

### 4.2. Sample Preparation

Harvested Tomato fruits were stored at −80 °C. Each batch of Micro-Tom was freeze-dried separately in a CryoneXt lyophilizer (Orlando, FL, USA).

### 4.3. Minerals Content

Fruits were dried at 65 °C for around two weeks. Afterwards, fruits were ground into a powder in a mortar. Around 10 mg of materials were weighted for each sample; the powder was then digested with 250 μL of 30% H_2_O_2_ as well as 750 μL of 65% nitric acid in 15 mL Digestion Cups (VWR, Radnor, PA, USA) and the tubes were degassed overnight. On the following day, the samples were incubated in the HotBlock (OnBoard, Meylan, France) for 8 h at 85 °C. After cooling, 4 mL MilliQ water was added and the samples were transferred to a 16 mm OD polypropylene tube (Agilent Technologies, Santa Clara, CA, USA). Mineral content of each sample was analyzed in technical triplicates with the 4100 MP-AES (Microwave plasma atomic emission spectrometry, Agilent Technologies, Santa Clara, CA, USA)

### 4.4. Macronutrients

#### 4.4.1. Carotenoids

Extraction procedures and conditions for analysis were performed as follows. Freeze-dried tomato samples were weighed (50 mg) with 300 mg of sand in 15 mL tubes. The tomato samples were rehydrated with 1 mL of distilled water and homogenized. Then, 10mL of a solution of Ethanol/Hexane (4:3, *v*/*v*) containing 0.1% BHT was added. The mixture was homogenized using a Vortex and then a Fast Prep^®^ 24 type agitation/grinding was applied at a speed of 6 m/s for 3 × 50 s. The hexane phase was collected in a 15 mL conical bottom falcon tube. The mixture was re-extracted twice in a row with 5 mL of hexane and Fast Prep 2 × 50 s. The hexane phases were then evaporated to dryness under nitrogen. Finally, the residue was re-dissolved in 500 μL of methyl tert-butyl ether (MTBE)/methanol (80:20 *v*/*v*) and 500 μL of dichloromethane and placed in an amber vial prior to HPLC analysis.

Carotenoid identification was performed on a reverse-phase HPLC DAD Agilent 1100 system (Agilent, Santa Clara, CA, USA) with a diode array detector. Carotenoids were separated using a C30 column (250 × 4.6 mm i.d., 5 μm) (YMC EUROP GmbH, Dinslaken, Germany) with a guard column, and the mobile phase was H_2_O as eluent A, methanol as eluent B, MTBE as eluent C. Operation temperature was set at 25 °C. The flow rate was set at 1 mL/min and the injection volume was 20 μL. A solvent gradient was programmed as follows: 0–2 min, isocratic 40% A—60% B (initial conditions); 2–5 min, 20% A—80% B; 5–10 min, 4% A—81% B—15% C; 10–60 min, 4% A—11% B—85% C; 60–70 min, isocratic 4% A—11% B—85% C; 70–71 min, 100% B; 71–72 min, with a return to the initial conditions for rebalancing. All-E-β-carotene and its isomer were detected at 450 nm, and all-*E*-lycopene and its isomers were detected at 470 nm. Isomers were identified according to their relative retention times, i.e., elution order and the combined use of their spectral data. The identifications were based on previously published data obtained with the same mobile phase (water/methanol/MTBE) and the same detection wavelength range.

#### 4.4.2. Sugars

Simple soluble sugars were analyzed by HPLC. Briefly, 500 mg of sample were rehydrated with 2 mL of distilled water and homogenized. Tomato samples were extracted three times with 10 mL ethanol (80%), then the mixture was heated at 70 °C for 10 min. After agitation for 20 min and centrifugation (4000× *g*, 5 min, 15 °C, Beckman Coulter, Brea, CA, USA), the supernatant was filtered through a 0.45 μm membrane before injection in UPLC. Samples were analyzed using a UPLC–1290 System Infinity II (Agilent, Santa Clara, CA, USA) equipped with a refractometer detector. A SHODEX SH1011 column 300 × 8 mm (Tokyo, Japan) was used with an isocratic system of water with H_2_SO_4_ (0.01%) and a flow rate of 0.7 mL/min. Temperature was set at 30 °C, injection volume at 10 μL, and spectrophotometric detection at 210 and 245 nm. External calibration was established for each standard sugar for concentrations from 0 to 10 g/L.

#### 4.4.3. Lipids

Lipid’s content was analyzed by the Folch method. A total of 500 mg of freeze-dried tomato samples were extracted three times with 15 mL of chloroform/methanol (2:1) solution. The mixture was agitated for 2 h, then centrifuged (6000 rpm, 10 min, 15 °C, Beckman Coulter). The supernatants were combined, then evaporated to dryness using a vacuum evaporation system (GeneVac EZ-2, SP Scientific, Warminster, PA, USA), and the tubes were weighed for the second time to determine the lipid content.

#### 4.4.4. Proteins

Protein content was calculated from nitrogen content assessed by the Kjeldahl method (Tecator Kjeltec) using a 6.25 conversion factor.

### 4.5. Evaluation of Antioxidant Bioactive Potential

#### 4.5.1. Total Polyphenol Content

Total polyphenols assay was performed with Folin–Ciocalteu reagent according to the method of Morel et al. [44]. Extracts of Micro-Tom and of rosemary (*Rosmarinus Officinalis*) were prepared in DMSO at 4 mg/mL and then diluted in water to be tested at a concentration of 1 mg/mL. A calibration curve was generated on a concentration range of 1.56 to 75 μg/mL of gallic acid. In a 96-well plate, 50 μL of extract, 50 μL of gallic acid, and 50 μL of distilled water were distributed in triplicate. Then 50 μL of 10% Folin–Ciocalteu reagent and 50 μL of sodium carbonate solution (1 M) were added. After 60 min in the dark, the absorbance was measured on a microplate reader (Molecular Devices, San Jose, CA, USA) at a wavelength of 650 nm. Results were expressed as milligrams of gallic acid equivalents (GAE) per gram of Micro-Tom extract.

#### 4.5.2. ORAC (Oxygen Radical Absorbance Capacity) Assay

The ORAC assays were performed in 96-well opaque polypropylene plates as previously described [44]. Samples were solubilized in DMSO at a concentration of 1 mg/mL before being diluted to 25 μg/mL using phosphate buffer at pH 7.4. On the 96-well microplate, 20 μL of Trolox solutions at 0.6, 25, 12.5, 25, 50 and 75 μM as standard curve, or chlorogenic acid (0.01 mg/mL), or ethanolic extract of rosemary (12.5 μg/mL) as a positive control, or the extracts at a concentration of 25 μg/mL, were applied. Then 100 μL of phosphate buffer and 100 μL of extemporaneously prepared fluorescein solution (0.1 μM in phosphate buffer) are added. The microplate is incubated at 37 °C for 10 min with shaking. The reaction was initiated with 50 μL of AAPH. Fluorescence was recorded at an excitation wavelength of 485 nm and an emission wavelength of 535 nm, for 70 min using a Tristar LB 941 microplate reader. Final ORAC values were calculated using a regression equation between Trolox concentration and area under the curve of decreasing fluorescein. Data are expressed as μmoles of Trolox equivalents per gram of dry extract.

#### 4.5.3. DPPH (2,2-Diphenyl-l-Picrylhydrazyl) Assay

Antioxidant activity was evaluated using the DPPH assay according to the method of [44]. Extracts were solubilized in DMSO (4 mg/mL) before being diluted in absolute ethanol to reach a concentration of 1 mg/mL. A standard curve of Trolox was performed (75, 50, 25, 12.5 μM). Ethanol was used as blank, and ethanolic extract of rosemary (0.2 mg/mL) and chlorogenic acid (0.01 mg/mL) were used as positive controls.

In a 96-well plate, 100 μL of positive control or extract were placed in each well. The test was performed in triplicate for each extract. A total of 75 μL of absolute ethanol and 25 μL of extemporaneously prepared DPPH solution (0.4 mg/mL) were introduced into each well. The plate was incubated for 30 min at room temperature and protected from light. The absorbance was read at 550 nm with a microplate reader (MDS Inc., Toronto, ON, Canada). Results were expressed as the mean plus or minus standard error to the mean of three independent experiments and were expressed as Trolox equivalents (TE μmoles per gram of dry extract). Results were also expressed as a percentage of inhibition (% inhibition) and calculated as follows: % inhibition = (OD blank − OD extract/OD blank) × 100.

### 4.6. Immunomodulatory Anti-Inflammatory Activity on Macrophage Cells Culture

Anti-inflammatory activity was assessed on the control regime (+F/+N) under both ambient CO_2_ and eCO_2_ conditions.

#### 4.6.1. Macrophage Culture

The macrophage cell line J774.A1 (ATCC, TIB67) was obtained from LGC Standards (Manchester, NH, USA). Cells were cultured in RPMI 1640 GlutaMAX^®^ medium supplemented with streptomycin (100 μg/mL) and penicillin (100 units/mL), 10% inactivated fetal calf serum (complete RPMI medium), cells were incubated at 37 °C, 5% CO_2_ and 95% humidity.

#### 4.6.2. Cell Viability Assay

To test cytotoxicity, 6.10^5^ cells/well were seeded in a 96-well culture plate in complete RPMI medium and incubated at 37 °C with different concentrations of extracts (25, 50, 75, and 100 μg/mL) for 20 h. After incubation, 20 μL/well of (3-(4,5-dimethylthiazol-2-yl)-5-(3-carboxymethoxyphenyl)-2-(4-sulfophenyl)-2*H*-tetrazolium), MTS, mixed with an electron coupling reagent, PMS in HBSS, was added. The plate was incubated for an additional 4 h and the absorbance at 490 nm was measured in a microplate reader (Molecular Devices, San Jose, CA, USA) as previously described [45].

#### 4.6.3. Dosage of NO (Nitric Oxide), PGE-2 (Prostaglandin E2), IL-6 (Interleukin-6), and TNFα (Tumor Necrosis Factor Alpha)

J774.A1 cells were seeded on a 24-well culture plate with complete RPMI medium. They were pretreated with various concentrations of Micro-Tom extracts of 100, 75, 50, 25 μg/mL for 4 h and stimulated with LPS (100 ng/mL) and interferon γ (10 ng/mL), and incubated for another 16–18 h at 37 °C. Supernatants were collected for nitrite determination or stored at –80 °C until use for PGE2, TNFα, and IL-6 dosages.

##### Determination of Nitrites (NO)

The presence of nitrite, a stable oxidized product of nitric oxide, was determined in the cell culture media as previously described [44]. Briefly, 100 μL of supernatant were combined with 100 μL of Griess reagent in a 96-well plate, and incubated 10 min at room temperature. Nitrite concentration was determined by measuring absorbance at 550 nm and using a NaNO_2_ standard curve (1.56 to 100 μM). Results were expressed as a percentage of inhibition values.

##### Interleukin 6 (IL-6) Assay

IL-6 production by J774 cells was determined with the IL-6 ELISA-kit (Mouse IL6 ELISA; Thermo Fisher Scientific, Vienna, Austria) after pretreatment with Micro-Tom extracts at a determined concentration range (25, 50, 75, 100 μg/mL) for 18 h. The cells were stimulated with 100 ng/mL LPS (*Escherichia coli*, 555B5) and 10 ng/mL mouse INFγ for 4 h. IL-6 release in cell supernatants was tested according to the ELISA Kit instructions. The results for IL-6 as well as for all other pro-inflammatory cytokines are expressed as a percentage of inhibition values.

##### Tumor Necrosis Factor Alpha (TNF-α) Assay

The TNF-α assay was performed according to the instructions contained in the kit-ELISA (TNF alpha Mouse Uncoated ELISA kit; Thermo Fisher Scientific). After pretreatment with the different concentrations of Micro-Tom extracts for 3 h, the cells were stimulated with LPS 100 ng/mL (*E. coli*, 555B5) and mouse INFγ 10 ng/mL for 4 h. TNF-α release in cell supernatants was tested by sandwich enzyme-linked immunosorbent ELISA assay.

##### Prostaglandin E2 (PGE-2) Assay

The determination of prostaglandins E2 was performed by the competitive ELISA assay on culture supernatants after pretreatment with Micro-Tom extracts and subsequent activation of the cells with LPS/IFNγ using the commercial Cayman PGE2 ELISA KIT Monoclonal (Cayman Chemical, Ann, Arbor, MI, USA).

### 4.7. Statistical Analysis

Statistical significance was assessed by one-way ANOVA followed by post hoc Tukey test or *T*-tests (significance was labeled as: *, *p* < 0.05 and **, *p* < 0.01).

## 5. Conclusions

In conclusion, the present study explored, for the first time, the impact of elevated atmospheric carbon dioxide concentration (eCO_2_) on the dwarf tomato *Solanum lycopersicum* L. cv. Micro-Tom nutrition-health properties. Taken all together, our results suggest several conclusions. First, Micro-Tom being considered a tomato model system, our investigations contribute to feed to the growing set of data characterizing this model for ongoing and future research on tomato. Micro-Tom reacting to eCO_2_ exposure as other C3 plants and tomato by higher carbohydrate content and lower protein content tends to consolidate its model status. Second, eCO_2_ exposure modifies mineral and carotenoid content and/or ratios suggesting plant metabolic adaptations. Third, biological activities are modulated by eCO_2_ exposure. The antioxidant effect is increased or decreased as a function of the scavenged free radical. Anti-inflammatory activity is modulated positively or unchanged as a function of the inflammatory marker recorded. Fourth, plants nutrients availability may influence the phytochemical response of the plant to eCO_2_ exposure and thereby its potential benefits for health.

Finally, several levels of complexity appear in the pursuit of a better understanding of the influence of eCO_2_ exposure on the nutrition health properties of tomato: (1) the complex interplay between eCO_2_ exposure and mineral nutrients available, as the latter also influence both plant chemistry and bioactivity, (2) the complex qualitative (type of molecules) and quantitative (molecular ratios) interplay between bioactive and/or non-bioactive compounds through synergistic and/or antagonistic interactions associated to “a large spectrum of molecular targets” [40] and (3) the complex interplay of the new bioactivity profile induced by eCO_2_ exposure and plant nutrients on the pathophysiological status of the consumers. Further in-depth investigations are needed [28] to explore these different research questions to gain insight into the real consequences of atmospheric CO_2_ evolution on agro-food-health systems and food crops, such as tomato, phytochemistry, and nutrition-health profile variations on the health of healthy and pathological populations. This is of particular importance in the context of climatic change and of growing demand for healthy food and prophylaxis of human non-communicable diseases.

## Data Availability

Not applicable.

## Sample Availability

Samples of the compounds are not available from the authors.
